# The differences in level of trait anxiety among girls and boys aged 13–17 years with myopia and emmetropia

**DOI:** 10.1186/s12886-016-0382-2

**Published:** 2016-11-14

**Authors:** Joanna B. Łazarczyk, Beata Urban, Beata Konarzewska, Agata Szulc, Alina Bakunowicz-Łazarczyk, Ewa Żmudzka, Urszula Kowzan, Napoleon Waszkiewicz, Karolina Juszczyk-Zajkowska

**Affiliations:** 1 Department of Psychiatry, Medical University of Bialystok, Brodowicza 1 16-070, Choroszcz, Poland; 2 Department of Pediatric Ophthalmology and Strabismus, Medical University of Bialystok, Waszyngtona 17, 15-274 Białystok, Poland; 3 Psychiatric Hospital of Choroszcz, Brodowicza 1, 16-070 Choroszcz, Poland; 4 Department of Psychiatry Faculty of Health Sciences, Medical University of Warsaw, Prof. Jan Mazurkiewicz Mazovia Specialist Health Centre, Partyzantów 2/4, 05-802 Pruszków, Poland

**Keywords:** Anxiety trait, Adolescence, Myopia

## Abstract

**Background:**

A significant increase in myopia among children and teenagers can be observed all over the world. Yet at the same time, there is still an insignificant number of studies concerning this health problem.

The aim of this study was to assess the level of trait anxiety among myopic group of teenagers in comparison to teenagers with emmetropia, and to confirm whether the level of trait anxiety relates to age and gender.

**Methods:**

Two hundred thirty-nine students aged 13–17 years were included in the study. The study group comprised 114 persons with myopia (81 girls and 33 boys), while the control group comprised 125 persons without refractive error (79 girls and 46 boys). Volunteers completed a set of questionnaires including: personal data, State-Trait Anxiety Inventory for Children (STAIC) (13–14 year-olds), or State-Trait Anxiety Inventory (STAI) (15–17 year-olds). The trait anxiety subscales were thus analyzed.

**Results:**

Among younger adolescents (13–14 years of age) with myopia there was a significantly higher incidence of pathological intensification of anxiety as a constant trait. After taking into account the distribution of gender, there was a higher level of trait anxiety in the group of boys with myopia than in the control group aged 13–17 years and 13–14 years. There was also a higher level of trait anxiety detected in males than in females.

**Conclusions:**

Myopia may affect the level of trait anxiety among 13–14-year-olds. In both age groups of girls, a higher percentage of patients with high level of anxiety was discovered (≥7 sten), as compared to their peers without vision defects. Our results can contribute to a more accurate analysis of young teenagers’ psychological problems, especially among boys diagnosed with myopia.

## Background

Myopia is one of the most common eye disorders in the world with east Asia having one of the world’s highest myopia rates. The prevalence of myopia is 9.7 % in 7-year-old Chinese children, 43.8 % in 12-year-old children, and 72.8 % in 18-year-old teenagers [1]. In comparison, 13 % of Polish students aged from 6 to 18 years were myopic [2]. Recently, there has been a tendency of myopia towards higher prevalence, greater severity (≤ -6.0 diopters) and younger age of onset [3]. Scientific findings have shown a growing tendency for the occurrence of myopia among teenagers [4]. This may be caused by civilization changes requiring more near vision work (reading, writing, working on a computer). The Australian study proves that myopia is nearly twice as common among 12-year-olds now than it was among their peers 5 years ago [5]. High myopia, in particular, is a public health and economic challenge due to significant risk factor for other ocular diseases, including glaucoma, retinal detachment and finally blindness [3, 6, 7]. Therefore, great efforts have been undertaken to prevent myopia onset and progression.

Although myopia is generally a treatable disorder, it may significantly affect visual function and the quality of life. There may be practical difficulties associated with the wearing and maintenance of optical corrective devices, and limitations imposed on sport and career opportunities [8]. The financial aspect of requiring spectacles, contact lenses or surgical correction is also a factor. A study of 112 myopic patients aged 18–65 years in the United Kingdom showed that patients with high myopia reported that psychological, cosmetic, practical, and financial factors affected their quality of life [7]. In the study by Dias et al. 469 myopic children reported moderate to high levels of self-esteem at follow-up in the areas of scholastic and athletic competence, physical appearance, social acceptance, behavioral conduct, and general self-worth. Mean scores ranged from 2.87 (+/- 0.68) on athletic competence to 3.40 (+/- 0.56) on general self-worth. Self-esteem changed significantly (*p* < 0.05) over 3 years in the domains of scholastic competence, social acceptance, and physical appearance [9].

Numerous reviews and studies report that myopic persons tend to differ from non-myopic persons along personality dimensions such as introversion/extroversion, passivity/anxiety, and abstractness/practicality. In a review of the literature, Lanyon and Giddings concluded that myopic patients are more introverted, embarrassed, and egocentric, as well as less outgoing in their social relationships; they also tend to have fewer friends, prefer indoor to outdoor activities, and are willing to participate in intellectual activities more often than non-myopic persons [10]. Baldwin also concluded that there is a relation between myopia and introversion, self-confidence, and reflexiveness [11]. In the study by Kalkan et al. myopic patients were found to have statistically significant lower rates in the low-order traits of purposefulness, cooperativeness, empathy, helpfulness, and compassion when compared to normal patients [12]. Self-directedness (a high-order trait of character) showed statistically lower rates in participants with myopia when compared to those with myopic astigmatism. Additionally, myopic individuals showed statistically significant lower rates than those with hyperopia in congruent second nature. Besides, myopic-astigmatic participants revealed statistically significant higher rates than those with hyperopia and myopia in empathy and helpfulness respectively.

In contrast to studies showing significant differences in personality characteristics between myopic persons and non-myopic persons, other studies have suggested that myopia and personality are not associated [13–16]. In one such study the authors aimed to determine whether myopia and personality are associated, but after multivariate analysis they did not support the view that myopic persons are introverted or conscientious [14]. Another prospective study on university students with myopia, emmetropia and hyperopia suggests that personality profile and psychophysical stress do not play a primary pathogenetic role in myopia [15].

Due to the rapid rate of increase in the incidence of myopia, the determination of its influence on teenagers’ mental health becomes pertinent. Unfortunately, there are not much studies available on the subject. On the other hand, it was discovered that other eye diseases, such as amblyopia and strabismus, have a negative effect on the patients’ mental state. A higher rate of somatization, obsessive-compulsive disorder, interpersonal sensitivity, depression and anxiety were observed in people with amblyopia, as compared to the control group. Packwood and coworkers found that psychological problems associated with amblyopia may affect individual self-esteem, work, school and friendships [17]. Moreover, children wearing glasses and treated with occlusion felt victim to overt bullying at school [18]. Horwood et al observed that the feeling of being a victim, which started in the early years of life, may be related to psychosocial maladjustment, and may cause an increase in anxiety, feelings of depression, loneliness; it may also cause low self-esteem and behavioral problems. In these studies, boys were more likely to fall victim to bullying than girls, especially if they were physically weaker than their peers. Defects of vision such as strabismus or amblyopia, were associated with worse interpersonal relationships and low self-esteem. There are also only few studies that report psychosocial effects of wearing glasses and negative feelings associated with such therapy in children, especially girls [19–21].

Therefore, we have decided to investigate, whether myopia (like in the case of amblyopia and strabismus) can be associated with a higher occurrence of mental disorders, especially if it is related to anxiety. This situation could be caused by a chronic dysfunction of visual acuity requiring wearing glasses or contact lenses, thus affecting the young person’s functioning and their perception by their peers. Therefore, we have decided to pre-define an increase in trait anxiety, as a factor predisposing for the development of anxiety disorders among teenagers with and without myopia.

We have put forward the following research hypotheses. The first of them assumes a higher level of trait anxiety among myopic groups of teenagers when compared to people without refractive error. We have also assumed that, among myopic persons, a higher level of trait anxiety can be found in the younger group (13–14 years of age), due to puberty occurring at that age and identification with features typical of a given gender. As the risk of development of anxiety disorders is higher among females, we expected that the trait anxiety level will be higher among girls suffering from myopia than in the group of myopic boys.

The identification of relationships between a higher level of trait anxiety among myopic teenagers can help to define preventive actions protecting teenagers from severe mental disorders during young adulthood. If such associations would exist, the follow-up for young patients with myopia should include a psychiatric evaluation in order to identify patients who may benefit from additional psychological exploration and support. If necessary, they should be referred for psychiatric support. Our research, involving patients aged 13–17 years, (the first one to our knowledge which involves that kind of age group) should enable to determine the importance of early screening for trait anxiety in myopic patients.

## Methods

### Participants

Two hundred and thirty-nine students aged 13–17 years were included in the study. The study group comprised 114 people with myopia over minus 6.0 D – 81 girls and 33 boys, while the control group comprised 125 people with normal visual acuity, without refractive error – 79 girls and 46 boys. The study group consisted of patients recruited from the outpatient clinic of The Department of Pediatric Ophthalmology and Strabismus, Medical University of Bialystok, Poland, and among students of middle and secondary schools. The control group consisted of middle and secondary schools students with emmetropia. Patients were included to the study after informing their parents or legal guardians and obtaining their written consent. The study group and the control group were divided according to age into subgroups of 13–14-year-olds and 15–17-year-olds, and according to gender.

### Procedure

Volunteers filled in a set of questionnaires consisting of: personal data, State-Trait Anxiety Inventory for Children (STAIC) (13–14-year-olds), or State- Trait Anxiety Inventory (STAI) (15–17-year-olds). The trait anxiety subscales were taken into account accordingly. The questions included in the personal data sheet concerned the person’s age, gender, his/her school, whether he/she had a refractive error, and if so, what kind of defect it is, since when and how many diopters it was.

The research received approval from the University Ethic Committee, and adhered to the tenets of the Declaration of Helsinki. All participants’ parents signed informed consent forms. We certify that all applicable institutional and governmental regulations concerning the ethical use of human volunteers were followed during this research.

### Measures

The State-Trait Anxiety Inventory for Children (STAIC) is a research instrument for studying anxiety defined as temporary and conditioned by the situation state of an individual as well as anxiety defined as a relatively constant personality trait. The STAIC consists of two subscales, the first subscale measures state anxiety, the second measures trait anxiety. Internal compliance of both scales in the Polish language version is high, but the absolute stability is lower, especially regarding the state anxiety scale. Cronbach’s alpha for the trait anxiety scale used in this study is .86 in the group of 13–14-year-old girls and .87 in the group of 13–14-year-old boys. The theoretical accuracy of both scales has been confirmed in several studies: the STAIC scores fundamentally correlate positively with school anxiety and negatively with motivation for learning and school achievements. It is used in the screening diagnosis to identify children who may have difficulties in school functioning and in the individual diagnosis – when causes of school failure are diagnosed. The state anxiety scale additionally may be useful in experimental studies that need to record changes in anxiety intensification and the trait anxiety scale in identifying children with neurotic tendencies [22].

The State-Trait Anxiety Inventory (STAI) is a research instrument for studying anxiety defined as temporary and conditioned by the state of an individual as well as anxiety defined as a relatively stable personality trait. The Polish language version of STAIC consists of two subscales - the first subscale measures state anxiety, the second one measures trait anxiety. Internal compliance of both Polish-language scales is high, but the absolute stability is lower, especially regarding the state anxiety scale. Cronbach’s alpha for the trait anxiety scale is .83 in the group of girls and .82 in the group of boys. The theoretical accuracy of both scales has been confirmed in several studies: the STAIC scores correlate significantly with the scores of instruments measuring theoretical constructs similar to anxiety; the accuracy of the state anxiety scale has been additionally verified and confirmed in numerous experimental studies. It is used for screening and individual diagnosis [23].

The results were presented as mean values ± standard deviation when data were normally distributed, otherwise as median (Mdn) and interquartile range (IQR). The results for STAIC and STAI scales were returned as sten scores. A sten score indicates an individual’s approximate position (as a range of values) with respect to the population of values and, therefore, to other people in that population. The individual sten scores are defined by reference to a standard normal distribution. Sten scores (for the entire population of results) have a mean of 5.5 and a standard deviation of 2.

When comparing the two groups, for characteristics consistent with a normal distribution (evaluated with the Shapiro-Wilk test) Student’s t-test was used, and for those not consistent with this distribution the Mann-Whitney test was applied. When comparing more than two groups, the univariate analysis of variance with post-hoc Bonferroni test or the ANOVA Kruskal-Wallis test by ranks were used respectively followed by the Mann-Whitney test. The CHI^2^ test for independence was used when comparing qualitative characteristics of selected groups. The effect size was calculated by using the Cohen’s method. The significance level of *p* < 0.05 was assumed as statistically significant. The statistical package SPSS was used for the calculations.

## Results

After comparing the results of STAIC and STAI using the trait anxiety scales, there were no significant differences between the percentages of persons with high severity of trait anxiety (≥7 sten)) (22,8 % in group with myopia vs 17,6 % in comparative group) and the median sten values in a group of adolescents with myopia (*n* = 114, Mdn = 5; IQR = 3) and a comparative group (*n* = 125, Mdn = 5, IQR = 3, *p* = 0.266, U = 6537) These groups had a non-normal distribution.

However, among younger adolescents (at the age of 13–14 years) with myopia, there was a significantly (*p* < 0.05) higher incidence of high intensification of anxiety as a constant trait. The percentage of patients with a high level of trait anxiety (≥ 7 sten) among 13–14-year-olds with myopia (*n* = 46) amounted 30.4 % vs. 14.6 % of those found in the control group (*n* = 48) (*p* < 0.05, effect size = 0.45). The median sten score was also significantly higher *(p* = 0.005*,* U = 765, effect size = 0.60) among younger teenagers with myopia (*Mdn sten score = 6.0, IQR = 2*) than among those with normal visual acuity (*Mdn sten score = 6.0, IQR = 2).* These groups had a nonnormal distribution.

After taking gender into account, there was a significantly higher (*p* = 0.011, effect size 0.60, *F* = 0.33, *t* = 2.60) level of trait anxiety in the group of boys aged 13–17 years with myopia (*n* = 33, *M sten score* = 5.9, *SD* = 1.80) than in the control group (*n* = 46, *M sten score* = 4.41, *SD* = 2.15) A higher severity of trait anxiety (*p* = 0.021, *F* = 2.45, t = 2.40*,* effect size = 0.74) mostly affected the group of younger boys suffering from myopia (*n* = 20, *M sten score* = 5.9, *SD* = 1.80 vs. *n* = 23, *M sten score* = 4.43, *SD* = 2.15) in the younger control group of boys. Moreover, the younger age group of girls with myopia (*n* = 26) presented a higher percentage (*p* < 0.05) of people with a high level of trait anxiety (≥ 7 sten) in comparison to their peers with normal visual acuity [*n* = 25] (34.6 % vs. 12 %, *effect size* = 0.48) and to the older age group of girls with myopia [*n* = 55] (34.6 % vs. 14.5 %, *effect size* = 0.54).

In addition, when the groups of girls and boys with myopia were compared, we found, that there was a significantly higher (*p* = 0.04, effect size = 0.44) level of trait anxiety in males (*M sten score* = 5.7, *SD* = 2.19) than in females (*M sten score* = 4.77; *SD* = 2.09), while in the control group this ratio was not statistically significant (*girls M sten score* = 4.85, *SD* = 2.00 vs. *boys M sten score* = 4.41, *SD* = 2.15, *p* = 0.256, *F* = 0.93, t = 1.14).

Cronbach’s alpha for the STAIC trait anxiety scale in this study was 0.85 in the group of 13–14-year-old girls and 0.83 in the group of 13–14-year-old boys. Cronbach’s alpha for the STAI trait anxiety scale was 0.85 in the group of girls and 0.84 in the group of boys.

## Discussion

Trait anxiety is defined by Spielberger as a theoretical construct, that “is a motive or acquired behavioral disposition, that predisposes a person to perceive a wide range of objectively non-dangerous (physically or psychologically non-dangerous) circumstances as threatening to respond to these anxiety reactions disproportionate in intensity and magnitude of the objective danger” [24]. This definition emphasizes the academic nature of anxiety. Spielberger is presents the opinion that the formation of anxiety can be traced back to the early childhood, to the relationship between a child and his/her parents in this period, but especially to punishment situations. This definition also indicates the role of cognitive processes (perception of the situation) in the formation of anxiety personality [23].

Anxiety disorders and depressive disorders are among the most common disorders experienced by youth, and can later contribute to adult anxiety disorders [25]. Yokoi et al tried to determine the incidence of depression and anxiety disorders in patients with high myopia as well as the factors that would predict the development of psychiatric complications and their impact on vision-related quality of life. They examined 205 patients with pathologic myopia. Incidence of depression was 22.0 % and incidence of anxiety disorder was 25.9 %. Twenty-two to 26 % of highly myopic patients had psychiatric disorders which had a strong negative impact on their vision-related quality of life [26]. On the other hand, Rosanes evaluated patients either with or without refractive errors, using a Rorschach Test, and reported that both patients with myopia and hyperopia showed significantly less expression of non-specific anxiety and hostility in comparison to healthy subjects [27]. That study also found that the manner of expressing anxiety covertly in patients with myopia was a decrease in motor activity and in patients with hyperopia as an increase.

Regarding how psychological factors are related to refractive errors, Seitler hypothesized that myopia is a result of a defense mechanism to tension that makes extraocular muscles tighten the eyeball, which directly causes refractive errors [28]. Furthermore, tension causes a break in the separation-individuation process in which myopic patients undergo separation anxiety that results with their sense of inability to cope with the world. Interestingly, the author noted that patients with myopia exhibit significantly higher levels of castration anxiety as compared with normal-sighted individuals [28].

Angi et al. have found a higher level of anxiety and somatization in myopic university students, as compared to the control group [29]. This study made us to decide on investigating the level of anxiety among myopic adolescents. Our hypothesis involved a higher level of trait anxiety among myopic group of teenagers than among people with visual acuity within normal limits. We have also set out to find a higher level of trait anxiety in the younger group among myopic people (13–14 years of age) and in the group of myopic girls. We managed to partly confirm our preliminary hypotheses. An increase in the incidence of anxiety defined as a constant trait was found in the present study in 13–14-year-olds with myopia, especially in boys. This result is surprising as, in accordance with the literature, the occurrence of anxiety-related disorders is the same both in girls and in boys and such disorders begin to prevail in the female gender from the puberty period (2:1 to 3:1) [30–33]. This can be explained by the fact that eyeglass wearers more often fall victim to bullying than those with normal visual acuity, while boys experience harassment more often than girls especially if they are physically weaker than their peers [18, 34–37]. Confirmation of this hypothesis may be found in the results of Dias et al. who showed that girls wear contact lenses more often than boys. Moreover adolescents who use contact lenses have higher levels of self-esteem (in terms of social acceptance, athletic skills and behavioral skills) than those wearing glasses [9].

Victims of bullying suffer not only because of a stressful situations, but also because of removal to the margin of the group and their low status among their peers [38]. Such rejection by the peers and a feeling of weakness may be a source of strong discomfort, because they are connected with non-compliance with the model of masculinity, and additionally boys ask for help more rarely than girls in case of maltreatment [39]. In people experiencing bullying, especially in young men, there is a high level of anxiety, depression, psychosomatic symptoms, abuse of alcohol and other psychoactive drugs [34, 36, 40]. A high incidence of suicidal thoughts in these people and their intentional self-mutilation deserve special attention [40–44]. It has also been found that in their future lives victims of bullying are more likely to develop anxiety disorders, depression, low self-esteem, feeling of loneliness and behavioral disorders, as well as abuse of psychoactive drugs [45–49]. In the recent study, it has been confirmed that the risk of developing depression in case of this type of maltreatment increases among boys and not among girls [50].

A higher percentage of people with high intensification of trait anxiety was found in the younger age group (13–14-years old) with myopia, both among boys and girls. One can suppose that it is associated with the phenomenon of a decrease in bullying prevalence in proportion to age [51].

### Limitations of the study

Due to differences in the occurrence of myopia between the genders, we collected a larger group of girls than boys. This could have affected the results of our research. In the future, a larger group of boys should be included to confirm the results of this research.

In the discussion section, we refer to studies concerning bullying in a group of people, especially concerning boys wearing glasses [18, 34–36]. Unfortunately, we did not examine this influence in our study. In the future, questions concerning the type of vision correction should be taken into account (spectacles, contact lenses) and the feeling of being a harassment victim (verbal, physical) as a result of vision defects.

Additionally, we used a self-completion questionnaire, which may influence the results. Although the tools were adapted to the respondents’ age, it is still a subjective evaluation. The use of an independent and objective measure of teenage anxiety would make the results more reliable.

There are no studies which would assess relationships between myopia and mental disorders, especially anxiety-related ones. For this reason, we could not refer to literature data in our study.

## Conclusions

We have succeeded in partial confirmation of the preliminary hypotheses. Myopia significantly affects the level of trait anxiety among 13–14-year-olds. In both age groups of girls, we have observed a larger percentage of persons with a high anxiety level (≥ 7 sten), as compared to peers without any vision defects. A higher anxiety level in the group of myopic boys, both as compared to peers of the same gender and as compared to myopic girls, was surprising. A higher level of trait anxiety among young people with lower visual acuity may result from the social perception of people wearing glasses as being weaker. Thus, they might more often become bullying victims. This results in deteriorated functioning, and may result in the development of anxiety and depression disorders, as well as abuse of psychoactive drugs.

Despite the fact that our results require a deeper analysis, they may provide some insight into the mental problems of younger teenagers, especially boys diagnosed with myopia. It is aimed at improving the quality of their lives and preventing the development of mental disorders resulting from a high level of anxiety. Additionally, it may contribute to a reduction of bullying behaviors towards them.
